# Cathodal tDCS Guided by Thermography as Adjunctive Therapy in Chronic Migraine Patients: A Sham-Controlled Pilot Study

**DOI:** 10.3389/fneur.2020.00121

**Published:** 2020-02-21

**Authors:** Giorgio Dalla Volta, Sara Marceglia, Paola Zavarise, Fabio Antonaci

**Affiliations:** ^1^Brescia Headache Center, Istituto Clinico Città di Brescia, Brescia, Italy; ^2^Dipartimento di Ingegneria e Architettura, Università degli Studi di Trieste, Trieste, Italy; ^3^Pavia Headache Center, C. Mondino National Institute of Neurology Foundation, IRCCS, University of Pavia, Pavia, Italy

**Keywords:** transcranial direct current stimulation (tDCS), frontal thermography (FIT), chronic migraine, cold patch, neuromodulation

## Abstract

**Objective:** To explore the efficacy of cathodal tDCS applied ipsilateral to the cold patch, as determined by thermographic evaluation, in the treatment of chronic migraine.

**Background:** Transcranial direct current stimulation (tDCS) is a non-invasive and safe technique that modulates the activity of the underlying cerebral cortex. tDCS has been extensively tested as a possible treatment for chronic pain and migraine with controversial results mainly due to the different setting procedure and location of electrodes. Since the presence of a hypothermic patch region detected through thermography has been suggested as a possible support for headache diagnosis, this “cold patch” could considered as possible effective location for tDCS application.

**Methods:** Forty-five patients with chronic migraine were randomized to receive either cathodal (25 patients) or sham tDCS, for 5 consecutive daily sessions plus a recall session after 1 month. Cathodal tDCS was delivered at 1.5 mA for 15 min in each session. Subjects were evaluated before treatment (baseline, T0), and after 10 (T10), 60 (T60), and 120 (T120) days after treatment. The number of attacks, duration of attacks, pain intensity, number of days with headache, and number of analgesics were collected at each time evaluation.

**Results:** Patients in the tDCS group showed a significant improvement compared to the sham group, during the whole study period in the frequency of migraine attacks (tDCS vs. sham: −47.8 ± 50.1% vs. −14.2 ± 16.5%, *p* = 0.004), number of days with headache (tDCS vs. sham: −42.7 ± 65.4% vs. −11.3 ± 18.0%, *p* = 0.015), duration of attacks (tDCS vs. sham: −29.1 ± 43.4% vs. −7.5 ± 17.6%, *p* = 0.016), intensity of the pain during an attack (tDCS vs. sham −31.1 ± 36.9% vs. 8.3 ± 13.5%, *p* = 0.004), and number of analgesics (tDCS vs. sham −54.3 ± 37.4% vs. −16.0 ± 19.6%, *p* < 0.0001).

**Conclusion:** Our results suggest that cathodal tDCS is an effective adjuvant technique in migraine provided that an individual correct montage of the electrodes is applied, according to thermographic investigation.

## Introduction

Finding the “right” preventive treatment for migraine often remains a challenge in many patients. Unlike triptans or gepans, which were designed to treat acute migraine attacks, the drugs currently used in migraine prophylaxis are not migraine-specific. Moreover, they are not devoid of side-effects and their efficacy rarely exceeds 50–60% at best (1). Chronic migraine patients (defined as having at least 15 days of headache per month, of which at least 8 migraine attacks) represent almost the 2–3% of the general population and their response to existing preventive therapies is often unsatisfactory (2, 3). Thus, treatments with better efficacy and tolerability are needed for migraine prophylaxis: these should ideally be disease-specific, i.e., designed to counteract the biochemical dysfunctions known to be involved in migraine pathogenesis.

It has been known for a long time that the brain excitability is abnormal in migraine during the interictal period (4). In particular, recent data suggest that migraine is characterized, at the network level, by metaplasticity-like mechanisms that, grounding on abnormal cortical excitability, lead to defective long-term potentiation (LTP) and long-term depression (LTD) mechanisms (5). For this reason in the last decade there has been an increasing interest for non-invasive brain stimulation (NIBS) techniques inducing excitability changes for migraine treatment (6, 7), which have been shown to produce metaplasticity-like patterns in migraine patients (5) and in visual cortex and associative areas (8).

Transcranial Direct Current Stimulation (tDCS) consists of applying direct current (DC) over the scalp using electrodes enclosed in perforated sponge pockets soaked with a saline solution or rubber electrodes with conductive gel (9). Depending on the size and polarity of the conducting electrodes, current intensity, density, and duration of the stimulation, it is able to induce changes in cortical excitability (10, 11). Long-lasting changes in the excitability of the motor cortex have been confirmed in humans (12, 13) and tDCS has been successfully studied in depression, chronic pain, stroke, Alzheimer disease, Parkinson's disease, and several other neurologic and neuropsychiatric conditions (9). The electrode positioning is critical for determining the direction and spatial distribution of the current flow and, ultimately, the effectiveness of the treatment (14).

Despite the heterogeneity of the results (15), tDCS has been extensively tested as a possible treatment for chronic pain (9). Being the effects both site-dependent and polarity-dependent, tDCS has been applied with different montages and different treatment sessions. Both monocephalic (active electrode on the scalp, reference electrode on the shoulder) and bicephalic (both electrodes on the scalp) were used, in different locations, such as anodal stimulation on V1 (16), anodal stimulation on M1 (17), frontal anodal stimulation (18), anodal stimulation on chronic pain affected patients (19). The available evidence on migraine patients is currently limited by the number of subjects treated (7, 17, 20–22) In addition, both single-application settings and multiple-application settings were tested, thus introducing another bias that prevents from a full comparability between the study results (15).

Despite the encouraging results, the most effective positioning for tDCS electrodes has still to be determined, and, provided the subjective nature of migraine, it is likely that a personalized, instead of standardized, electrode positioning, could improve outcomes.

A possible indication on the best electrode positioning for tDCS may come from thermography that involves the use of an infrared camera to portray the body surface temperatures, that might be a possible support for headache diagnosis (23). In fact, a preliminary work on 60 patients with migraine, cathodal tDCS applied over the cold patch was able to significantly reduce frontal hypothermia at 3 months follow-up (21). In addition, we recently suggested a standardized procedure for the definition of the cold patch, an asymmetric thermic pattern observed in the frontal area of migraine patients (24–26), that can provide thermographic parameters discriminating between migraine patients and controls (23).

Based on this concept, in a preliminary study, the authors demonstrated that cathodal tDCS applied ipsilateral to the frontal hypothermic patch region reduces the cold patch thus being a potentially effective prophylactic therapy in migraine patients (21).

In the present study, according to our preliminary observations that cathodal stimulation ipsilateral to the hypothermia determines a reduction of the cold patch, we explored the efficacy of this montage as addon treatment in chronic migraine.

## Methods

### Patients and Experimental Protocol

Forty-five patients (15 M, and 30 F, mean age 45 ± 3.7) with chronic migraine (from at least 6 months), with or without medication overuse, defined according to the International Headache Society [ICHD-3 beta criteria (27)] were recruited in the outpatient clinics of the Headache Center of Istituto Clinico Città di Brescia (Brescia, Italy) and the Pavia Headache Center (Mondino Hospital, Pavia, Italy).

On the enrolment visit, the diagnosis was confirmed, and a thorough neurological examination was performed. Inclusion criteria were: ages between 18 and 65 years; migraine without aura; migraine present for at least 6 months before the enrolment. Exclusion criteria were: history of acute neurological, psychiatric or medical disease; family history of epilepsy; pregnancy or lactation; cardiac pacemaker; skull defects; previous surgery involving implants in the head; any central of peripheral neurostimulation treatments before enrolment; any previous preventive therapies for migraine.

The study was approved by the institutional review board (Ethical Committee of the Fondazione IRCCS Istituto Neurologico Nazionale Casimiro Mondino, date of approval: July 29th 2013) and conformed with the Declaration of Helsinki. All patients signed written informed consent before participating to the study.

All subjects were treated with topiramate, titrated in 20 days' time up to the therapeutic dosage of 50 mg bid. After baseline examination (T0), subjects were randomly assigned to receive either cathodal or sham tDCS. They were blind to the type of tDCS they received and they were aware of the fact that they might receive either sham or real stimulation. They were instructed to fill in a daily diary to record onset and duration of headache attacks, pain intensity (on a scale from 1—slight pain—to 10—worst conceivable pain), assumption and response to analgesics. Onset of headache attacks following the main environmental inducing factors were monitored throughout the study, in order to estimate the sensitivity to migraine triggering in each patient. After treatment, patients were monitored data were collected at day 10 (T10), 60 (T60), and 120 (T120). The following variables were extracted from diary reports:

- Number of attacks (monthly average)
- Duration of attacks (monthly average)
- Number of monthly days with headache (primary endpoint of this pilot study)
- Number of analgesics (monthly average)
- Pain intensity (monthly average).

### tDCS

**Figure 1:**
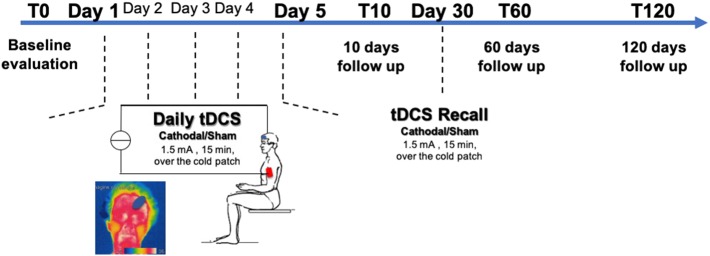
Experimental protocol—Transcranial Direct Current Stimulation (tDCS) was applied unilaterally on the frontal cortex over the cold patch as determined by thermographic examination for 5 consecutive days. A recall session was then performed at 30 days. Patients were randomized to receive either cathodal tDCS or sham tDCS. Patients were assessed at baseline (T0), after 10 days (T10), after 2 months (T60), and after 4 months (T120) from the end of tDCS treatment.

tDCS was delivered in 5 consecutive daily sessions and 2 recall sessions after 1 month (Figure 1). Direct current was transferred by a saline-soaked pair of surface sponge electrodes (5 × 7 cm) and delivered by a battery-driven CE marked constant current stimulator (HDCkit—Newronika srl). A constant current of 1.5 mA intensity was applied for 10 min, with an applied charge density of 25.71 mC/cm^2^. A monocephalic stimulation montage was used in which the active electrode (cathode) was placed over the cold patch as identified by thermographic examination, using the standard protocol previously described (23). In brief, thermographic images were captured through an infrared thermal camera (model LT3, Zhejiang Dali Technology Co. Ltd.) with a thermal sensitivity <0.08°C at 30°C. The subject was placed 1 meter far from the camera in a room under standard condition. The cold patch was identified as the coolest point in the forehead.

For sham stimulation, the electrode montage was the same, and the stimulation device was turned ON for 30 s before ramping down to 0, so that the subject could feel the typical itching sensation at the electrode site occurring at the beginning of the stimulation. This made sure that the subjects could not discriminate between sham or real stimulation.

### Data Analysis

All variables (number of attacks, duration of attacks, pain intensity, number of days with headache, and number of analgesics) were analyzed independently. We first verified that the two groups (tDCS, sham) had comparable baseline values using an independent-samples *t*-test (*p* < 0.05).

Then, we verified whether cathodal tDCS ipsilateral to the cold patch was effective over time. To do so, we calculated the percentage change from baseline of each variable considered at each time point, according to the formula:

$$\begin{array}{l} {\text{D}}_{\text{Tx}}=\left({\text{V}}_{\text{Tx}}-V_{B}\right)/{\text{V}}_{\text{B}} \end{array}$$

Where D_Tx_ is the percentage change at the time Tx (T10, T60, or T120), V_Tx_ is the value of the considered variable V at the time Tx, and V_B_ is the value of the considered variable at baseline (T0). The effect of tDCS was investigated through a two-way mixed model analysis of variance (ANOVA) with factors time (within factor, 3 levels: T10-T60-T120) and stimulation (between factor, 2 levels: tDCS, sham). Tukey's Kramer *post-hoc* test was then applied to include possible effects driven by multiple comparisons (*p* < 0.05).

In addition, we further refined this analysis on the raw values of each variable through a two-way mixed model analysis of variance (ANOVA) with factors time (within factor, 4 levels: T0-T10-T60-T120) and stimulation (between factor, 2 levels: tDCS, sham) and with Tukey's Kramer post-hoc test (*p* < 0.05).

## Results

tDCS (*N* = 28) and sham (*N* = 17) group were comparable in terms of frequency of migraine attacks (16.5 ± 12.2 vs. 11.3 ± 5.9, *p* = 0.06), number of days with headache (20.8 ± 10.1 vs. 15.5 ± 8.0, *p* = 0.06), duration of attacks (22.9 ± 22.8 vs. 22.4 ± 16.9, *p* = 0.91), intensity of the pain during an attack (7.0 ± 1.5 vs. 7.9 ± 1.4, *p* = 0.06), and number of analgesics (15.3 ± 10.5 vs. 17.0 ± 9.4, *p* = 0.06). At baseline, 16 patients in the tDCS group and 10 in the sham group were in medication overuse.

tDCS was applied according to the position of the cold patch (15 subjects had the cold patch on the right side, and 13 on the left side).

**Figure 2:**
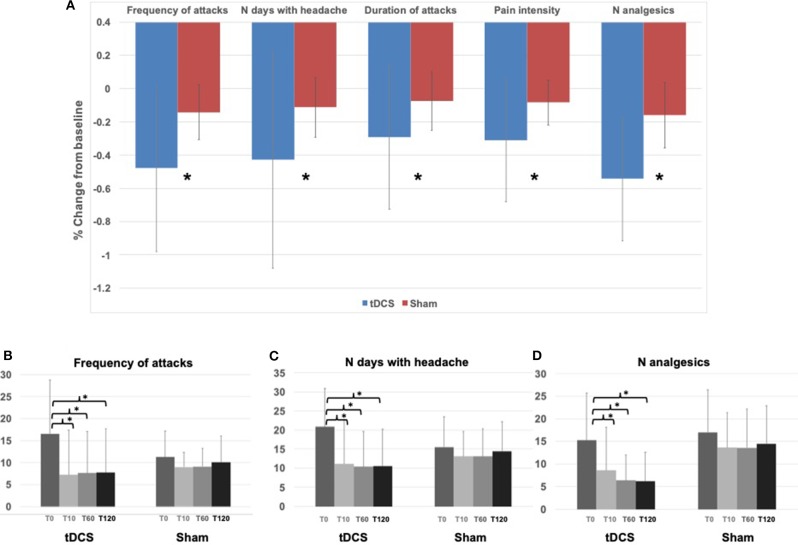
Results—**(A)** Histograms represent the average (across all patients and all time points, T10, T60, T120) percentage change from baseline (T0) in the tDCS and Sham groups for all the variables assessed. * indicates ANOVA factor “stimulation” *p* < 0.05. **(B)** Histograms represent the average frequency of attacks in the tDCS and Sham group at T0, T10, T60, and T120. * indicates Tuckey's honest *post-hoc* test for the ANOVA interaction “time x stimulation,” *p* < 0.05 (T0 vs. T10 *p* = 0.00003; T0 vs. T60 *p* = 0.00003; T0 vs. T120 *p* = 0.00003). **(C)** Histograms represent the average number of days with headache in the tDCS and Sham group at T0, T10, T60, and T120. * indicates Tuckey's honest Post-hoc test for the ANOVA interaction “time x stimulation,” *p* < 0.05 (T0 vs. T10 *p* = 0.00003; T0 vs. T60 *p* = 0.00003; T0 vs. T120 *p* = 0.00003). **(D)** Histograms represent the average number of analgesics in the tDCS and Sham group at T0, T10, T60, and T120. ^*^ indicates Tuckey's honest Post-hoc test for the ANOVA interaction “time x stimulation,” *p* < 0.05 (T0 vs. T10 *p* = 0.00003; T0 vs. T60 *p* = 0.00003; T0 vs. T120 *p* = 0.00004).

In general, patients in the tDCS group showed a significant improvement in all variables analyzed compared to the sham group, during the whole study period (Figure 2A). In fact, a significant effect of the factor “stimulation” in the ANOVA comparison between percentage changes from baseline was observed in the frequency of migraine attacks (tDCS vs. sham: −47.8 ± 50.1% vs. −14.2 ± 16.5%, *p* = 0.004), number of days with headache (tDCS vs. sham: −42.7 ± 65.4% vs. −11.3 ± 18.0%, *p* = 0.015), duration of attacks (tDCS vs. sham: −29.1 ± 43.4% vs. −7.5 ± 17.6%, *p* = 0.016), intensity of the pain during an attack (tDCS vs. sham −31.1 ± 36.9% vs. 8.3 ± 13.5%, *p* = 0.004), and number of analgesics (tDCS vs. sham −54.3 ± 37.4% vs. −16.0 ± 19.6%, *p* < 0.0001). The effects of time and of the interaction “time x stimulation” were not significant.

**Figure 3:**
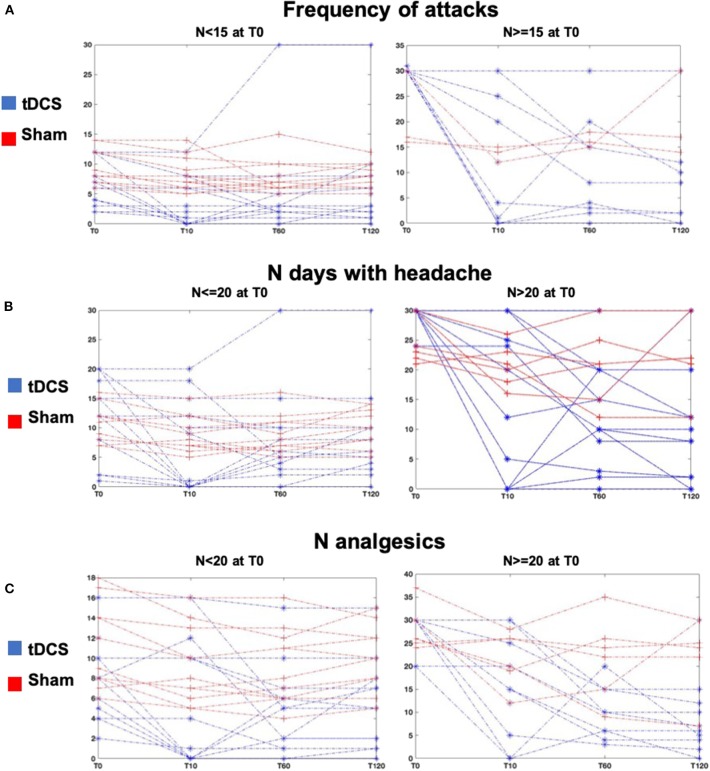
Results—**(A)** Lines represent the individual values of frequency of attacks of all patients at all time points, T10, T60, T120 in the tDCS (blue lines) and Sham (red lines) groups. The left plot shows patients with frequency of migraine attacks <15 at T0, and the right plot shows patients with frequency of migraine attacks ≥15 at T0. **(B)** Lines represent the individual values of number of days with headache of all patients at all time points, T10, T60, T120 in the tDCS (blue lines) and Sham (red lines) groups. The left plot shows patients with number of days with headache ≤20 at T0, and the right plot shows patients with number of days with headache >20 at T0. **(C)** Lines represent the individual values of number of analgesics of all patients at all time points, T10, T60, T120 in the tDCS (blue lines) and Sham (red lines) groups. The left plot shows patients with number of analgesics <20 at T0, and the right plot shows patients with number of analgesics ≥20 at T0.

The analysis of the time course of tDCS effects compared to sham showed that there was a consistent and significant decrease only in the tDCS group of the frequency of migraine attacks (ANOVA interaction “time x stimulation” *p* = 0.006, Figures 2B, 3A) of the number of days with headache (ANOVA interaction “time x stimulation” *p* = 0.001, Figures 2C, 3B), and of the number of analgesics (ANOVA interaction “time x stimulation” *p* < 0.0001, Figures 2D, 3C). As shown in Figure 3, patients in the tDCS group experienced a marked and consistent reduction in all the variables, despite the value at baseline, whereas patients in the Sham group reported more stable values across time.

Patients did not report any adverse event during the experiment. None complained specific tDCS-related side effects. Topiramate was well-tolerated in all patients, without any mild or severe adverse effects reported during the observation period.

## Discussion

In the present study we explored the efficacy of cathodal tDCS applied ipsilateral to the hypothermia for 5 consecutive days as adjuvant treatment in chronic migraine, and demonstrated that this technique provides beneficial effects up to 120 days after treatment ended, in terms of duration, intensity, and frequency of attacks.

Our results are in line with previous encouraging observations reporting a reduction in the consumption of analgesics and triptans, with a perception of increased efficacy of the therapy, after a session of 10 anodal tDCS over M1 (2 mA, 20 min), performed twice a week in average (28). Other previous positive results were reported by Dasilva et al. (17) that, for the first time, described the application of a similar protocol (10 anodal tDCS in 1 month, over M1, cathode on the contralateral supraorbital area) at an intensity of 2 mA for 10 sessions in a month. In another contribution (20), 13 patients were treated with cathodal stimulation of V1 for 9 sessions (3 times per week, 1 mA) with a current of 1 mA showing a small reduction of the intensity and average duration of the attacks, but the attack frequency did not change. Anodal stimulation of the dorsolateral prefrontal cortex (12 sessions, 2 mA, 20 min) resulted more effective than M1 and sham tDCS in a recent three-arm study (22), even though the low number of subjects (3 patients in the DLPFC arm) limits the strength of the conclusion. Our work adds a repeatable personalized protocol providing beneficial effects up to 120 days after stimulation cycle.

As far as we know, this is the first study using tDCS as a prophylactic treatment in migraine with a patient-specific montage, guided by the thermography. Reduced pain triggering threshold and altered regulation of cutaneous vasoconstriction in migraine might be two different aspects of a hyperexcitable neural network. Indeed, cathodal tDCS is believed to reduce cortical excitability in the area underlying the electrode (29, 30), probably by reducing intracortical facilitation and increasing intracortical inhibition (10, 30, 31). By activation of non-synaptic mechanisms (32), cathodal tDCS may be able to induce hyperpolarization of neuronal membranes and modulate neuronal firing leading to long-lasting firing rate depression. In addition to the reduction of headache attacks intensity and frequency, patients under treatment experienced a reduced sensitivity to environmental trigger factors: a further evidence of modulation of the neuronal excitability induced by tDCS.

Since migraine is associated with abnormal neuronal excitability between attacks, we also hypothesized that inhibitory tDCS might be effective in migraine prophylaxis by diminishing interictal cortical excitability, with a selective effect of cathodal tDCS that may last beyond the end of stimulation (33). This could be also explained by the priming effect of cathodal tDCS on cortical excitability in migraine patients reported by (5) which was not shown in healthy controls, suggesting that cathodal tDCS may help reversing the altered pattern of short-term homeostatic plasticity characterizing migraineurs. The prospect that plastic changes in neural network underlie the pathogenesis of chronic pain partially elucidate why different pharmacological modalities are only able to obtain modest relief. Long-term modulation of cortical plasticity can be achieved non-invasively by tDCS, thus obtaining long-lasting therapeutic effects.

Pinchuk et al. (18) already demonstrated that the effectiveness of tDCS in different types of headache depends on the right localization of the stimulating electrode, but they did not characterize which is the most promising electrode positioning. Our results indicate that cathodal tDCS applied over the frontotemporal cortex homolateral to the cold patch, individuated by thermography, is effective in reducing the frequency of migraine attacks (both as number of attacks and number of headache days per month), their duration (single attacks and total hours of headache per day), and the number of analgesics taken in a month. It has been hypothesized that the cold patch corresponds anatomically to the shunt between internal carotid artery circle (through the ophtalmic artery) and the external carotid artery (superficial temporal artery). This hypothermic area remains unchanged over time in patients with migraine and changes only after therapeutic interventions (26), thus suggesting that the cold patch could act as a useful prognostic marker, related to the clinical status and effectiveness of treatments. A disappearance of the cold patch was observed after successful administration of sumatriptan in acute attacks (34). The mechanism underlying the disappearance of the cold patch is supposed to involve the balance of sympathetic and parasympathetic system (i.e., reduction of sympathetic hypertonia and vasoconstriction of skin microcirculation). Even though it is not yet clear whether the cold patch is an epiphenomenon of migraine or if it is involved in its mechanisms, there is evidence that the thermic asymmetry is specific of migraine, and tends to disappear with effective treatments. These observations, together with the results of our pilot study, further corroborate the hypothesis that this protocol can be effectively applied as migraine adjuvant treatment.

Despite being generally in line with reported tDCS effects on migraine, our results show that cathodal, instead of anodal tDCS is effective. This difference could be due to the stimulation site, considering that this is the first work specifically targeting the cold patch. Also, we applied a extracephalic montage, instead of cephalic, which limits the confounding factors related to the stimulation of different brain areas (35). In addition, outside the motor cortex, the excitatory or inhibitory effects of anodal and cathodal tDCS are debatable (7).

tDCS is not the only NIBS technique applied in migraine. Repetitive transcranial magnetic stimulation (rTMS) was applied in large trials with encouraging results [for review, see (7)]. However, other potential targets may be taken into account, such as Non-invasive Vagus Nerve Stimulation [nVNS, (36)], Transcutaneous Supraorbital and Occipital Electrical Neurostimulation [tSNS and tONS, (37, 38)], as well as the cerebellum (39).

## Conclusions

In conclusion, our data suggest that the efficacy of tDCS in migraine is dependent on a correct montage of the electrodes, which must be customized to the single patient. Our tDCS protocol is a promising and safe therapeutic opportunity, deserving further studies in larger patients populations.

## Data Availability Statement

To ensure patient's confidentiality and privacy, the datasets generated for this study can be found in authors' repositories available upon request.

## Ethics Statement

The studies involving human participants were reviewed and approved by Ethical Committee of the Fondazione IRCCS Istituto Neurologico Nazionale Casimiro Mondino, date of approval: July 29th 2013. The patients/participants provided their written informed consent to participate in this study.

## Author Contributions

GD and FA ideated the research, designed the experimental protocol, coordinated and conducted experiments, and revised draft manuscript. PZ conducted the experiments and revised the draft manuscript. SM conducted data analysis, drafted the manuscript, and finalized writing.

### Conflict of Interest

SM is founder and shareholder of Newronika srl, a spin-off company of the Fondazione IRCCS Ca'Granda Ospedale Maggiore Policlinico of Milan and of the University of Milan. The remaining authors declare that the research was conducted in the absence of any commercial or financial relationships that could be construed as a potential conflict of interest.
